# Porcine gut loops to explore the impact of amino acids on the host–microbiota crosstalk in the ileum

**DOI:** 10.3389/fmicb.2026.1854850

**Published:** 2026-06-05

**Authors:** Fanny Renois, Caroline Hervet, Céline Barc, Nathalie Kasal-Hoc, Elisabeth Jones, Charlie Roux, Dominique Gagnon, Tristan Chalvon-Demersay, Martin Beaumont, Francois Meurens

**Affiliations:** 1 Research Group on Infectious Diseases in Production Animals (GREMIP) and Swine and Poultry Infectious Diseases Research Center (CRIPA), Faculty of Veterinary Medicine, University of Montreal, Saint-Hyacinthe, QC, Canada; 2 Oniris, INRAE, BIOEPAR, Nantes, France; 3 INRAE Centre Val de Loire, UE-1277 Plateforme d’Infectiologie Expérimentale (PFIE), Nouzilly, France; 4 GenPhySE, Université de Toulouse, INRAE, ENVT, Castanet-Tolosan, France; 5 METEX ANIMAL NUTRITION, Paris, France

**Keywords:** amino acids, gut loops, intestinal barrier, microbiota, pig

## Abstract

Amino acids (AAs) are essential for the growth, development, and health of pigs, yet their direct effects on the intestinal barrier and microbiota remain poorly understood *in vivo*, despite extensive *in vitro* studies on mucosal barrier integrity. While intestinal cell lines and organoids provide valuable insights, they cannot fully capture the complexity of the *in vivo* intestinal mucosa, where diverse cell types interact dynamically with each other and the microbiota. To address this gap, we hypothesized that the ligated intestinal loop model could serve as an intermediate system, clarifying the effects of AAs on the intestinal mucosa and microbiota while reducing the need for experimental animals. This study aimed to test this surgical approach for the first time, assessing the short-term impact of AA administration and laying the groundwork for future model optimization. We prepared ten ileal loops in four weaned pigs and administered either PBS, lysine (3 g/L), a mixture of branched-chain AAs (3 g/L), or tryptophan (3 g/L) for 48 h. Subsequent analyses of loop content metabolites, microbiota composition, and mucosal gene expression revealed that gene expression, metabolic, and microbial profiles were primarily driven by interindividual variability among pigs. Although no statistically significant differences were detected between treatment groups, several trends were identified. While the intestinal loop model shows potential for short-term assessments of AA mixtures on the intestinal mucosa and microbiota, further optimization is needed to fully realize its potential.

## 1 Introduction

Amino acids (AAs) are fundamental to the growth, development, and health of animals, including pigs (Darmaun, 2008; Ling et al., 2023; Fayomi et al., 2025). Their diverse roles extend beyond being building blocks for proteins. In the gut, AAs serve as vital regulatory molecules, influencing key physiological processes, such as epithelial barrier function, digestion, immune fitness, oxidative stress homeostasis, and microbiota balance (Wang et al., 2009; Mou et al., 2019; Yang and Liao, 2019; Chalvon-Demersay et al., 2021; Beaumont et al., 2022b). More specifically, AAs play a critical role in maintaining the structural integrity of the mucosal barrier, particularly by modulating tight junction proteins essential for epithelial cohesion (Chalvon-Demersay et al., 2021; Beaumont et al., 2022b). Research indicates that dietary L-Tryptophan enhances the expression of zonula occludens-1 (ZO-1) and occludin, proteins fundamental to tight junction integrity, thereby improving intestinal permeability (Liang et al., 2018). Additionally, specific AAs such as isoleucine and leucine have demonstrated the capacity to support and restore epithelial barrier function in rotavirus infection models (Mao et al., 2015, 2018), particularly by enhancing mucin production in the jejunal mucosa of weaned piglets (Mao et al., 2015). Then, dietary supplementation with specific AAs, such as lysine, can modulate the expression of AAs and glucose transporters in pigs (He et al., 2013). AA can influence also gut health through their involvement in metabolic signaling pathways (Wang et al., 2009; Ling et al., 2023). The phosphoinositide 3-kinase/protein kinase B/mammalian target of rapamycin (PI3K/AKT/mTOR) pathway, which is vital for cell growth and metabolism (Tang et al., 2018). Leucine activates the mTOR signaling pathway, which regulates protein synthesis and cell proliferation in the gut (Corl et al., 2008; Mao et al., 2015). These signaling interactions affect the growth and repair of intestinal cells and play a role in immune responses. This is crucial for maintaining intestinal integrity and recovery after injury or infection.

The gut microbiota composition and functionality are significantly influenced by dietary AAs, further affecting host gut health. AAs can selectively modulate microbial communities (Beaumont et al., 2022b). For instance, BCAA supplementation can increase the abundance of bacteria such as *Lactobacillus* and *Coriobacteriaceae* while reducing bacteria like *Ruminiclostridium* and *Clostridiales_vadinBB60* (Hu et al., 2019; Beaumont et al., 2022b). These modulations are associated with the production of AA-derived metabolites, which effects are either protective or detrimental, mostly depending on their concentration (Beaumont et al., 2022b; Van den Abbeele et al., 2022; Fang et al., 2025). BCAA are metabolized by gut bacteria into branched-chain fatty acids (BCFA), such as isovalerate, isobutyrate, and 2-methylbutyrate. These metabolites serve as energy sources for colonocytes and help maintain gut barrier function (Beaumont et al., 2022b; Fang et al., 2025; Beaumont et al., 2026). Tryptophan, metabolized by gut bacteria into bioactive compounds such as indole derivatives and tryptamine, enhances microbial diversity and selectively promotes the growth of bacteria—including *Prevotella*, *Roseburia*, and *Succinivibrio*—while reducing bacteria such as *Clostridium sensu stricto* and *Clostridium XI* (Liang et al., 2018; Beaumont et al., 2022b). Moreover, tryptophan derived metabolites can enhance gut barrier function by reducing epithelial permeability and inflammation (Roager and Licht, 2018). Additionally, the polyamines cadaverine and 5-aminovalerate are produced through microbial decarboxylation of lysine but the consequences for the gut mucosa remain unclear (Oliphant and Allen-Vercoe, 2019).

The effects of AA supplementation on growth performance in pigs underscore their critical role in gut development, particularly during the perinatal period and at weaning (Mou et al., 2019). Enhanced dietary formulations that incorporate functional AAs have proven effective in reducing post-weaning stress and improving gut morphology, characterized by increased villus height and crypt depth, indicators of enhanced nutrient absorption capacity (Zong et al., 2018; Xiong et al., 2019). The contributions of AAs to structural integrity of the gut, cellular signaling, microbiota modulation, and improved growth performance designate AA as a crucial component that warrants further exploration to optimize pig health.

Numerous studies to clarify the impact of AAs on the intestinal mucosa have been conducted using cell lines (Wang et al., 2014; Kong et al., 2018), intestinal organoids (Beaumont et al., 2022a), or after oral administration of AAs to pigs (Deng et al., 2023; Pearce et al., 2024; Koo et al., 2025). Intestinal cell lines and intestinal organoids are highly useful, but they do not fully capture the complexity of an *in vivo* intestinal mucosa, where multiple cell types interact together and with the microbiota. Conversely, precisely identifying the effects of AAs on the intestinal mucosa and the mechanisms involved after oral administration is challenging due to the system’s high complexity. In the present study, we hypothesize that the ligated intestinal loop system (Gerdts et al., 2001; Meurens et al., 2009; Girard-Misguich et al., 2011; Bayne et al., 2025) could serve as an intermediate model to clarify the impact of AAs on the intestinal mucosa and microbiota and help elucidate certain mechanisms. Indeed, the ligated intestinal loop model (Gerdts et al., 2001) is an *in vivo* surgical method used to study localized mucosal responses in the small intestine of various mammals including sheep (Gerdts et al., 2001) and pigs (Meurens et al., 2009; Girard-Misguich et al., 2011). This method allowed researchers to analyze immune responses, gene expression, or microbiota in a controlled environment while preserving normal tissue function. The key advantages of this procedure include the ability to test multiple treatments within a single animal, reducing variability and the need for additional subjects. The ligated intestinal loop model preserves intact blood and lymphatic connections, ensuring physiological relevance. It can also be adapted to include or exclude microbiota, depending on research objectives. This versatility makes it ideal for studying mucosal immunity, vaccine efficacy, and the role of Peyer’s patches in immune induction (Griebel and Hein, 1996; Mutwiri et al., 1999), to our knowledge, it has not yet been applied to assess the specific impact of AAs on the intestinal mucosa and microbiota. To test our hypothesis, we injected individual AA or AA mixtures into unwashed ileal loops and analyzed their effects. Specifically, we evaluated: (1) the expression of transcripts associated with the intestinal barrier and AA transporters; (2) the composition and diversity of the microbiota; (3) metabolite concentrations. The primary goal of this study was to pioneer the use of this surgical approach, assessing the short-term impact of AA administration while laying the groundwork for its further optimization.

## 2 Materials and methods

### 2.1 Animals

The study adhered to the ARRIVE guidelines (https://arriveguidelines.org) and was conducted at INRAE (PFIE, Nouzilly, France) in compliance with Directive 2010/63/EU regarding animal experimentation. The experimental protocol received approval from the Regional Ethical Committee in Animal Experiment of Tours (CEEA VdL, committee number 19, France) and the French Ministry of Higher Education, Research and Innovation (authorization APAFIS #37059-2022050312008665 v4). Four female pigs of Piétrain × Large-White × Landrace lineage, bred from a single sow at INRAE UEPAO, were selected for the experiment and were weaned at 28 days of age, 7 days before the surgery. Intestinal loop surgery is a relatively invasive and complex procedure. Using four animals for an initial assessment of the impact of AAs on the intestinal mucosa was both logistically reasonable and relevant, providing sufficient sample size for preliminary statistical evaluations with a small cohort. Moreover, the different treatments could be compared within the same pig.

### 2.2 Gut loop surgery and AA inoculation

**Figure 1:**
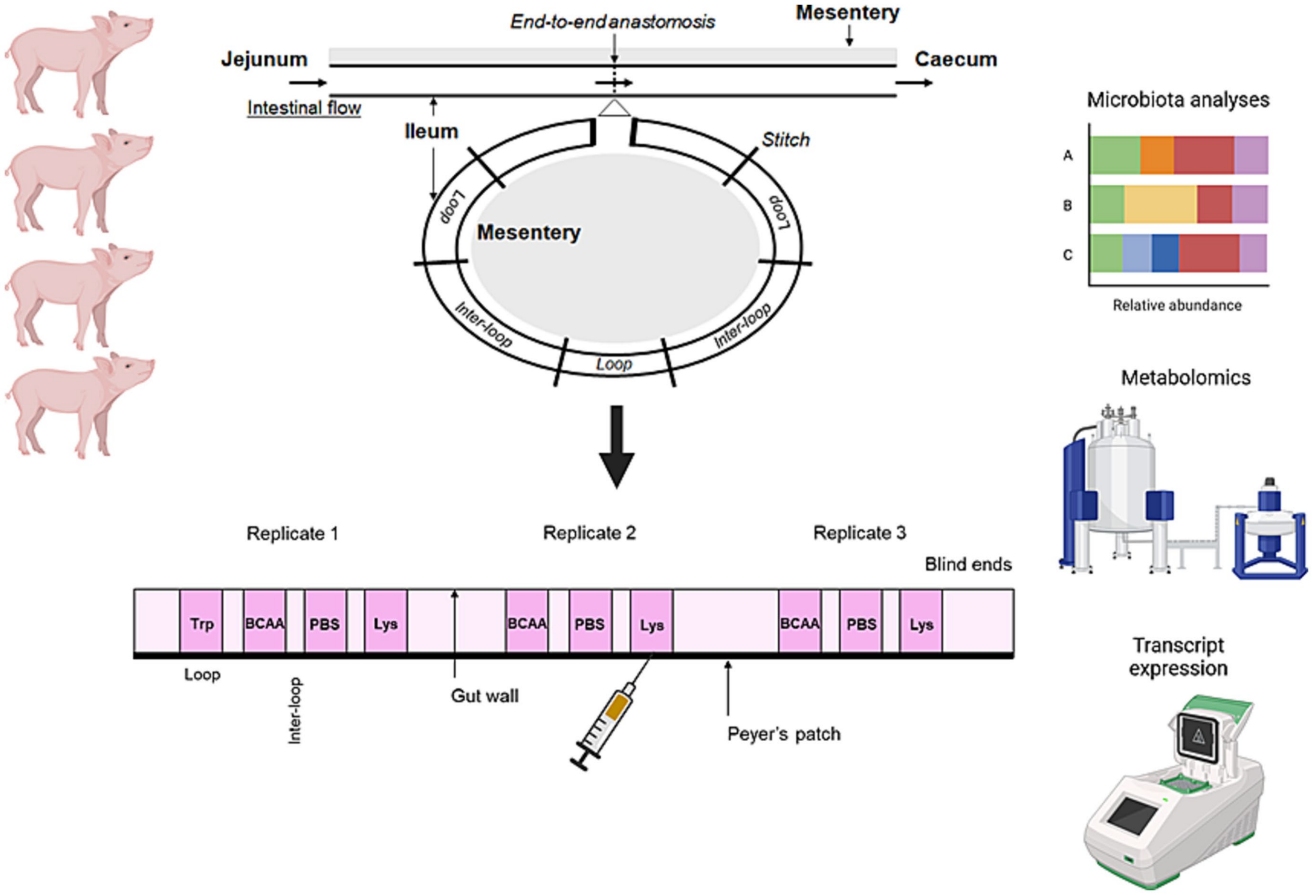
Schematic representation of the ileal loops. A total of ten ileal loops were created in each of the four pigs. AAs [5 mL - 3 g/L in PBS - L-lysine (LYS), a mix of branched chain AAs (BCAA) including L-valine, L-leucine, and L-isoleucine (1:1:1) or L-tryptophan (TRP)] or phosphate-buffered saline (PBS) devoid of AA were injected into the loops. Pigs were maintained 48 h after AA injection before euthanasia, the collect of the samples and subsequent analyses. Created in BioRender. Meurens (2025) https://BioRender.com/2hxpra3.

To create the loops, a sterile 2-m segment of the intestine was surgically prepared in the ileum. This surgical procedure was performed on four pigs weaned 7 days before over the course of 2 days [for detailed surgical procedures, refer to Gerdts et al. (2001) and see Figure 1 for a schematic of the ileal loops]. The ‘intestinal-segment’ was then divided into consecutive 5 cm sections, termed ‘loops’ (ten loops) and ‘inter-loops’ (nine inter-loops), which also contain PP (see Figure 1). The intestinal segments proximal and distal to the experimental segment are reconnected, restoring normal transit. The content of the “ileal-segment” was reduced since the piglets were fasted for 12 h before surgery. Therefore, it was not washed, which allowed the initial microbiota to be preserved. Then, AAs (5 mL - 3 g/L in PBS - L-lysine (LYS), a mix of branched chain AAs (BCAA) including L-valine, L-leucine, and L-isoleucine (1:1:1) or L-tryptophan (TRP)) (Sigma-Aldrich, St-Quentin Fallavier, France) or Phosphate-Buffered Saline (PBS, Dominique Dutscher SAS, Brumath, France) devoid of AA were injected into the loops using a 22G needle (Terumo) (see Figure 1). The dose of 3 g/L was based on a previous study (Miner-Williams et al., 2009). The published values of total AAs in ileal digesta, expressed on a dry matter (DM) basis, included approximately 13.3 mg lysine/g DM. Assuming an ileal digesta dry matter content of 10 to 20%, this value would correspond approximately to 1.3 to 2.7 g/L lysine in wet digesta. The selected dose of 3 g/L was therefore considered appropriate for an initial local stimulation experiment. For each treatment, we used three replicate loops, except for the L-tryptophan treatment, where only one loop per animal was created due to technical constraints (the ileum being shorter than the jejunum, where more loops can be created). Post-surgically, pigs were treated with 20 μg/kg intramuscularly (IM) three times/day Buprenorphine (Buprecare, Animalcare, Dunnington York, UK), a semi-synthetic opioid, for 2 days, and 0.6 to 1 mg/kg three times/day subcutaneous Ketamine (Imalgene, Merial, Lyon, France). Pigs were maintained 2 days (48 h) after AA injection. We selected a 48-h exposure period based on the technical feasibility of the ligated intestinal loop model. This duration allows sufficient time for potential changes in mucosal gene expression, microbial composition, and metabolite profiles to occur, while ensuring animal welfare post-surgery. Pigs were fed Sevryplus (Sanders SA, Paris, France) and water *ad libitum* and carefully monitored daily for abdominal discomfort, pain, body temperature, cardiac and respiratory frequency, and the transit of feces. Two days after surgery, the pigs were euthanized via intracardiac injection of pentobarbital (60 mg/kg) (Vetoquinol, Lure, France) following sedation with xylazine (2 mg/kg) and ketamine (20 mg/kg) (intramuscular) (Vetoquinol and Merial). The loops were collected during the necropsy. For transcript expression analysis, tissues were dissected into five 3 × 3 mm sections including the continuous Peyer’s patch (Peyer’s patch sample) or not (gut wall sample). Then, the samples were rinsed with ice-cold phosphate-buffered saline, snap-frozen in liquid nitrogen, and stored at −80 °C. The content of each loop was collected, snap-frozen in liquid nitrogen, and stored at −80 °C until metabolomics and microbiota analyses.

### 2.3 Immune transcript expression analysis in the ileal mucosa

To detect transcripts related to immune and AA responses, real-time PCR primers were designed and optimized using Clone Manager 9 (Scientific & Educational Software, Cary, NC, USA) and procured from Eurogentec (Liège, Belgium), following established protocols (see Table 1 for the primer list). Ileal tissue samples (around three mm^3^ pieces) were homogenized in Trizol reagent (Invitrogen, Cergy Pontoise, France) with ceramic beads (BioSpec Products, OK, USA), and total RNA was extracted using the RNeasy Plus Mini Kit (Qiagen, Courtaboeuf, France). RNA concentration was determined by measuring the optical density at 260 nm (OD260), and RNA quality was assessed by calculating the OD260/OD280 ratio and using Nanophotometer (Implen, Munich, Germany). Complementary DNA (cDNA) was synthesized from 600 ng of using GoScript™Reverse Transcriptase (Promega, Charbonnières-les-Bains, France) as per the manufacturer’s protocol. The cDNA was diluted 3X and combined with primer sets (0.2 μL of 10 mM primer stock) and GoTaqqPCR Master mix (Promega) according to the manufacturer’s instructions. The real-time PCR was executed on a CFX96 Bio-Rad OPUS system (Bio-Rad, Hercules, CA, USA) with cycling conditions set to an initial denaturation cycle at 95 °C for 2 min, followed by 40 cycles of amplification at 95 °C for 3 s and 58 °C to 67 °C for 30 s, depending on the gene of interest (see Table 1 for details). Fluorescence was measured automatically throughout the PCR assay. The CFX Maestro 2.3 software (Bio-Rad) was used to determine the quantification cycle (C*q*) for each reaction. A melting curve analysis was performed for each primer pair to confirm the specificity of amplified products, checking for a single peak. qPCR assays were conducted as previously described (Saade et al., 2020), utilizing three of the most stable reference genes for normalization, namely Beta-2-microglobulin, Ribosomal protein L4, and TATA box binding protein. The qPCR data were expressed as relative values using Genex macro analysis (Bio-Rad) (Vandesompele et al., 2002).

**Table 1:** Primer sequences and qPCR conditions for gene expression analysis in porcine ileal mucosa.

Primer abbreviation and full name	Primer sequences: sense (S) and anti-sense (AS)	Amplicon size (bp)	Annealing temperature (°C)	Efficiency (%)	Accession number or reference
AID *Activation-induced cytidine deaminase*	(S) AGAAGTTTCAAAGCCTGGGAG (AS) TCAACCTCATACAGGGGCAAA	92	57	94–103	Hervet et al. (2025)
APRIL/TNFSF13 *A proliferation-inducing ligand*	(S) TGCTCACCCGTAAACAGAAG (AS) TAAACTCCAGCATCCCAGAC	172	60	92	Meurens et al. (2009)
(B2M1) *Beta-2-microglobulin*	(S) CAAGATAGTTAAGTGGGATCGAGAC (AS) TGGTAACATCAATACGATTTCTGA	161	58	91	Nygard et al. (2007)
BAFF/TNFSF13B *B-cell activating factor*	(S) GGAGACGGTCCCCATCCT (AS) AGCAGCTTCCCATCTTTGGA	69	60	109	NM_001097498.1
CAT-1	(S) CATCAAAAACTGGCAGCTCA (AS) TGGTAGCGATGCAGTCAAAG	185	60	90–110	He et al. (2013)
CCL20 *Chemokine (C-C motif) ligand 20*	(S) GCTCCTGGCTGCTTTGATGTC (AS) CATTGGCGAGCTGCTGTGTG	146	65	90	Meurens et al. (2009)
CCR9 *Chemokine (CC motif) receptor 9*	(S) TACGGCTATGACGCCACACC (AS) ACGGCACCCACGATGAACAC	143	69	95	Meurens et al. (2009)
CCR10 *Chemokine (CC motif) receptor 10*	(S) TCCTGCTTTCTGCAGCTCTC (AS) GGGTGGAAAGGTGTGGAATG	185	64	109	Larcher et al. (2019)
GP2 *Glycoprotein 2*	(S) GGCCTGCCTTCCTCATTCAT (AS) TACAAATTCACCTCCCGGCC	123	62	99	XM_005662102.3
IL6 *Interleukin 6*	(S) ATCAGGAGACCTGCTTGATG (AS) TGGTGGCTTTGTCTGGATTC	177	60	106	Meurens et al. (2009)
IL8/CXCL-8 *Interleukin 8*	(S) TCCTGCTTTCTGCAGCTCTC (AS) GGGTGGAAAGGTGTGGAATG	100	62	92	Meurens et al. (2009)
NLRP6 *NOD-like receptor family pyrin domain containing 6*	(S) GGCTTCTCGGACAAGGACAA (AS) GCACAGAGAGAACAGCGTCT	114	60	94	XM_021082529.1
OCLN *Occludin*	(S) TTGTGGGACAAGGAACGTATTTA (AS) TGCCTGCCGACACGTTT	76	60	97	Metzler-Zebeli et al. (2018)
(RPL4) *Ribosomal protein L4*	(S) CAAGAGTAACTACAACCTTC (AS) GAACTCTACGATGAATCTT	122	60	97	Nygard et al. (2007)
SLC6A14–ATB^0,+^ *Sodium- and chloride-dependent neutral and basic amino acid transporter B(0+)*	(S) CCGTGGTAACTGGTCCAAAAA (AS) CCAATCCCACTGCATATCCAA	65	60	90–110	Sun et al. (2015)
SLC6A19–B^0^AT1 *Sodium-dependent neutral amino acid transporter B(0)AT1*	(S) CACAACAACTGCGAGAAGGA (AS) CCGTTGATAAGCGTCAGGAT	155	60	90–110	Sun et al. (2015)
SLC7A7–y^+^LAT1 *y + L amino acid transporter-1*	(S) GCCCATTGTCACCATCATC (AS) GAGCCCACAAAGAAAAGC	216	60	90–110	Sun et al. (2015)
SLC7A9–b^0,+^AT *b0,+ amino acid transporter*	(S) ATCGGTCTGGCGTTTTAT (AS) GGATGTAGCACCCTGTCA	145	60	90–110	Sun et al. (2015)
SOX8 *Transcription factor SOX-8*	(S) TCCAGCACAAGAAGGACCAC (AS) CGTCCGTCTTGTACACAGCA	135	63	88	XM_021086873.1
(TBP) *TATA box binding protein*	(S) AACAGTTCAGTAGTTATGAGCCAGA (AS) AGATGTTCTCAAACGCTTCG	153	60	93	Nygard et al. (2007)
TSLP *Thymic stromal lymphopoietin*	(S) AGGGCTTGTGCTAACCTAC (AS) ATCCGGCCTATCATCACAG	164	58	91	XM_021085155.1
ZO-1 *Zonula Occludens-1*	(S) AAGCCCTAAGTTCAATCACAATCT (AS) ATCAAACTCAGGAGGCGGC	130	60	107	Metzler-Zebeli et al. (2018)

The reference genes are between brackets.

### 2.4 16S rRNA gene amplicon sequencing and sequence analysis

DNA was extracted from 40 mg of ileal loop content using the Quick-DNA Fecal/Soil Microbe Miniprep Kit (ZymoResearch Europe GmbH, Freiburg, Germany) according to the manufacturer instructions. PCR amplicons of the 16S rRNA gene V3-V4 region were sequenced by MiSeq technology (Illumina France SARL, Paris, France) at the Genomic and transcriptomic platform (GeT-PlaGe, INRAE, Toulouse), as described before (Read et al., 2019). Sequencing reads were deposited in the National Center for Biotechnology Center for Biotechnology Information Sequence (accession number: PRJNA1308782). Amplicon sequences were analyzed by the FROGS pipeline version 4.0.1 (Escudié et al., 2018), following the guidelines. Merged sequences were selected based on their size (350–500 nucleotides), dereplicated and counted. Sequences were then clustered into OTUs by using Swarm (clustering aggregation distance: 1). After PCR chimera removal, the OTUs present in less than 3 samples or which proportion represented less than 0.005% of all sequences were filtered out. The taxonomic affiliation of OTUs was performed with the 16S SILVA database (138.1, pintail 100). A phyloseq object with OTU count table and sample metadata was created. The mean number of reads per sample was 25,819 (min: 17861 – max: 34836). For *α* and *β*-diversity analyses, the count table was rarefied to 17,861 sequences per samples with the R software (4.2.0) and the phyloseq package (1.40.0). Microbiota richness (number of observed OTUs) and Shannon and Inverse Simpson α-diversity index were calculated. β-diversity was analyzed with the distance of Bray-Curtis and visualized by non-metric multidimensional scaling (nMDS). The unrarefied count table was used to calculate the relative abundances of bacterial taxa at the phylum, family and genus level.

### 2.5 Nuclear magnetic resonance-based metabolomics

Loop content samples (50 mg) were homogenized in 500 μL phosphate buffer (pH 7, prepared in D_2_O, containing 1 mM TSP used as a chemical shift reference) with a FastPrep instrument and using tubes containing lysing D matrix (MP Biomedicals, Illkirch-Graffenstaden, France). After centrifugation (12,000 *g*, 10 min, 4 °C), the supernatant containing metabolites was recovered and the extraction step was repeated on the pellet. The two fractions of supernatant were pooled and then centrifuged twice (18,000 *g*, 10 min, 4 °C). The obtained supernatants (600 μL) were transferred to 5 mm nuclear magnetic resonance (NMR) tubes. All NMR spectra were obtained with an Avance III HD NMR spectrometer operating at 600.13 MHz for ^1^H resonance frequency using a 5 mm inverse detection CryoProbe (Bruker Biospin, Rheinstetten, Germany) in the MetaboHUB-MetaToul-AXIOM metabolomics platform (Toulouse, France). ^1^H NMR spectra were acquired at 300 K using the Carr-Purcell-Meiboom-Gill spin-echo pulse sequence with presaturation. Pre-processing of the spectra (group delay correction, solvent suppression, apodization with a line broadening of 0.3 Hz, Fourier transform, zero order phase correction, shift referencing on TSP, baseline correction, setting of negative values to zero) was performed in the Workflow4Metabolomics Galaxy tool following guidelines (Guitton et al., 2017). After water region (4.5–5.1 ppm) exclusion, spectra (0.5–9 ppm) were bucketed (0.01 ppm bucket width) and normalized by total area. For metabolite identification, spectra of pure compounds prepared in the same buffer and acquired with the same spectrometer were overlaid with sample spectra. For each identified metabolite, a bucket non-overlapping with other metabolites were selected for the quantification (Table 2).

**Table 2:** Identification of metabolites in porcine ileal loop content NMR spectra.

Metabolite	δ^1^H (ppm)
2-methylbutyrate	0.86 (t)*
Valerate	0.89 (t), 1.31 (m)*, 1.53 (m)
Butyrate	0.90 (t)*, 1.56 (m), 2.16 (m)
Isovalerate	0.92 (d)*
Isoleucine	0.94 (t)*, 1.01 (d)
Leucine	0.97 (t)*
Valine	1.00 (d), 1.05 (d)*
Propionate	1.06 (t)*, 2.19 (m)
Isobutyrate	1.07 (d)*
Doublet@1.15 ppm	1.15 (d)*
Ethanol	1.19 (t)*, 3.66 (m)
Lactate	1.33 (d)*
Alanine	1.48 (d)*
Lysine	1.73 (m)*, 3.03 (t)
Acetate	1.92 (s)*
Glutamate	2.06 (m), 2.13 (m), 2.36 (m)*
Succinate	2.41 (s)*
Methylamine	2.60 (s)*
Trimethylamine	2.88 (s)*
Creatine	3.04 (s)*, 3.93 (s)
Choline	3.20 (s)*
Phenylacetate	3.54 (s), 7.31 (t)*, 7.39 (t)
Glycine	3.57 (s)*
Uracil	5.81 (d)*, 7.55 (d)
Fumarate	6.52 (s)*
Tyrosine	6.91 (d)*, 7.20 (d)
Phenylalanine	7.34 (d), 7.38 (t), 7.43 (t)*
Hypoxanthine	8.22 (d)*

### 2.6 Statistical analyses

The R software (4.2.0) was used for statistical analyses. Microbiota, metabolomics and gene expression data were analyzed by linear mixed models (lme4 and car packages) after power of 0.25 (microbiota) or log transformation (metabolomics and gene expression), respectively. Models included the group as a fixed effect and the replicate and the pig as a random effect. Statistical analyses were performed only for bacterial taxa which relative abundance was over 0.5% within at least one group since this threshold was previously shown to ensure reproducible quantifications by 16S rRNA gene amplicon sequencing (Paës et al., 2020). PERMANOVA with 999 permutations was used to study the effects of group, replicate and pig on the microbiota structure (vegan package). Principal components analyses (PCA) were performed with the mixOmics package on the normalized gene expression and metabolomics data.

## 3 Results

All four pigs involved in the study recovered without any major complication following surgery, resuming normal feeding as early as the day after the procedure and maintaining good health until euthanasia. Pig 3, however, experienced a brief episode of post-operative diarrhea. Notably, no other clinical signs were observed throughout the study period.

**Figure 2:**
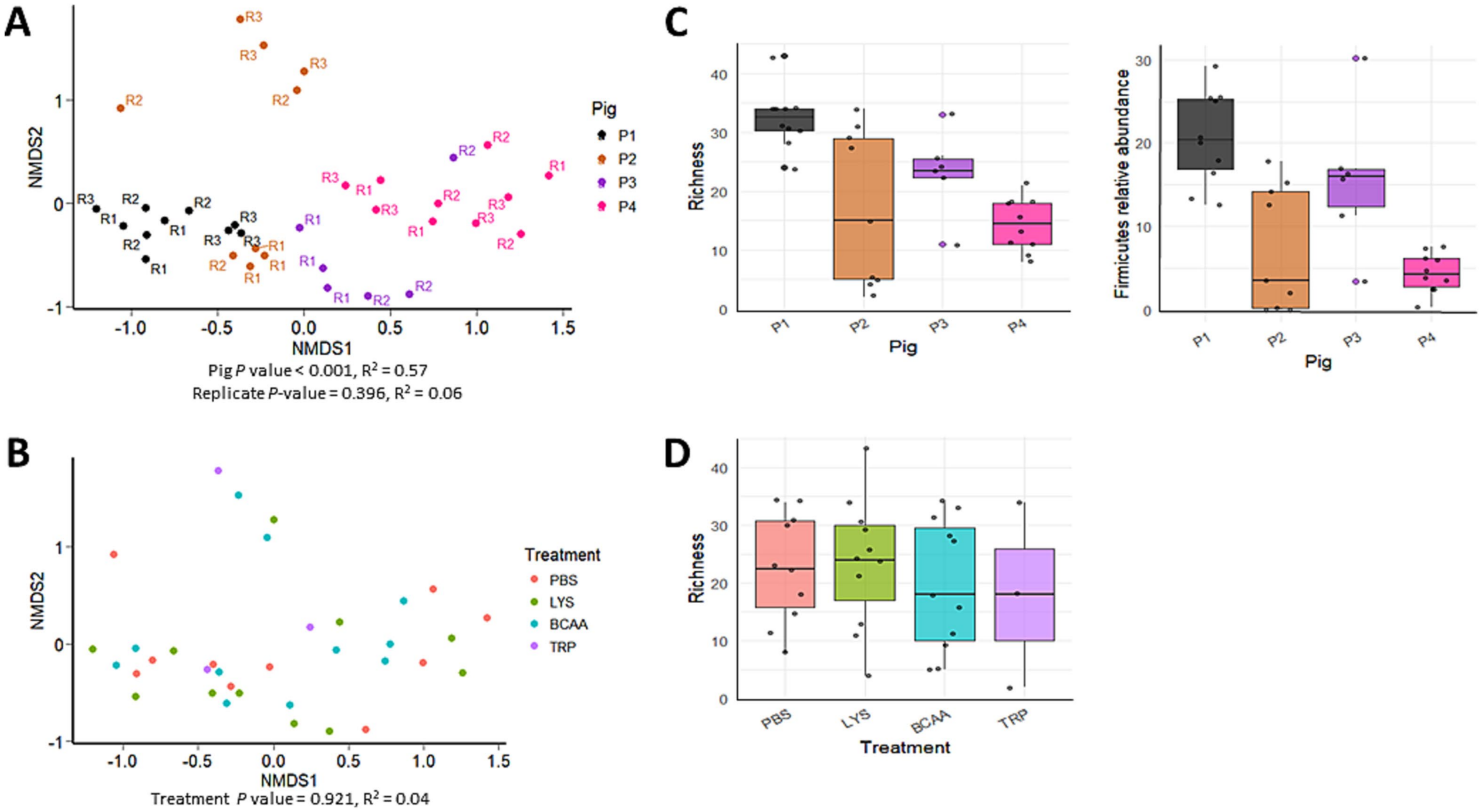
Ileal loop content microbiota. Non-metric dimensional analysis (NMDs) showed that the microbiota composition was primarily driven by individual pig and replicate effects **(A)**, whereas AA treatments had no significant impact **(B)**. The richness of microbial communities varied with treatment **(D)**, while the relative abundance of *Firmicutes* and overall richness differed among individual pigs **(C)**. BCAA: mix of branched chain AAs including L-valine, L-leucine, and L-isoleucine; LYS: L-lysine; PBS: phosphate-buffered saline; R: replicate; TRP: L-tryptophan.

The impact of treatments on the gut microbiota in ileal loop contents is presented in Table 3 and Figures 2A–D. Replicate 3 of Pig 3 was excluded from the analysis due to an atypical microbiota/metabolite profile, but the data are available in the supplementary figures (Supplementary Figure 1). All these observations presented in Table 3 and Figures 2A–D were trends and no significant differences were identified (*p* > 0.05) except for *Colidextribacter* genus, for which, nevertheless, we failed to identify pairwise differences between groups. Beta-diversity analysis of microbiota showed clustering by individual pigs (Figure 2A) but not by treatments (Figure 2B). For instance, Pig 1 exhibited the highest microbial richness and abundance of *Firmicutes* (Figures 2C,D). Overall, it was not possible to distinguish between the four treatments based on microbiota composition or richness. Of note, the ileal loop microbiota was dominated by the phylum *Proteobacteria* with genera such as *Escherichia-Shigella* and *Campylobacter*.

**Table 3:** Relative abundance of bacterial phyla and families in ileal loop microbiota.

	*p-*value	Pairwise comparisons				Relative abundance (mean)				Relative abundance (SEM)			
						PBS	LYS	BCAA	TRP	PBS	LYS	BCAA	TRP
Phylum													
*Firmicutes*	0.214	a	a	a	a	12.15	13.46	11.24	5.73	3.15	2.93	2.47	3.97
*Campylobacterota*	0.577	a	a	a	a	28.81	20.27	25.37	7.0	12.12	9.92	10.8	6.99
*Proteobacteria*	0.584	a	a	a	a	58.97	64.63	61.57	78.72	10.32	8.6	10.11	11.39
*Bacteroidota*	0.411	a	a	a	a	0.01	1.12	1.81	8.54	0	0.77	1.80	8.54
*Actinobacteriota*	0.951	a	a	a	a	0.03	0.52	0.01	0.02	0.03	0.52	0.01	0.02
Family													
*Clostridiaceae*	0.914	a	a	a	a	2.85	1.79	1.59	0.66	1.69	0.62	1.1	0.61
*Enterococcaceae*	0.437	a	a	a	a	8.79	10.07	7.57	4.92	2.22	2.91	1.62	3.25
*Campylobacteraceae*	0.577	a	a	a	a	28.81	20.27	25.37	7.0	12.12	9.92	10.8	6.99
*Pasteurellaceae*	0.506	a	a	a	a	26.95	25.62	17.67	6.18	5.21	7.19	3.82	3.99
*Enterobacteriaceae*	0.293	a	a	a	a	32.02	39.01	43.90	72.55	9.68	10.09	11.39	15.23
*Bacteroidaceae*	0.223	a	a	a	a	0	1.11	1.8	8.54	0	0.77	1.8	8.54
*Lachnospiraceae*	0.579	a	a	a	a	0.46	0.97	2.04	0.13	0.41	0.7	1.4	0.13
*Oscillospiraceae*	0.144	a	a	a	a	0	0.56	0	0	0	0.31	0	0
*Actinomycetaceae*	0.565	a	a	a	a	0	0.51	0	0	0	0.51	0	0
Genus													
*Clostridium sensu stricto 1*	0.59	a	a	a	a	2.39	0.99	1.1	0.02	1.63	0.53	1.0	0.02
*Enterococcus*	0.437	a	a	a	a	8.79	10.07	7.57	4.92	2.22	2.91	1.62	3.25
*Campylobacter*	0.577	a	a	a	a	28.81	20.27	25.37	7.00	12.12	9.92	10.8	6.99
*Pasteurella*	0.386	a	a	a	a	1.23	1.25	3.08	0.68	0.48	0.52	1.06	0.42
*Actinobacillus*	0.31	a	a	a	a	25.72	24.37	14.59	5.5	5.16	7.08	3.79	3.57
*Escherichia-Shigella*	0.215	a	a	a	a	32.02	33.90	39.13	72.55	9.68	10.49	12.01	15.23
*Bacteroides*	0.223	a	a	a	a	0	1.11	1.8	8.54	0	0.77	1.8	8.54
*Lachnoclostridium*	0.803	a	a	a	a	0.45	0.71	1.57	0.13	0.41	0.49	1.09	0.13
*Colidextribacter*	0.041	a	a	a	a	0	0.56	0	0	0	0.31	0	0
*Enterobacter*	0.551	a	a	a	a	0	1.3	1.22	0	0	1.3	1.22	0
*Actinomyces*	0.565	a	a	a	a	0	0.51	0	0	0	0.51	0	0

**Figure 3:**
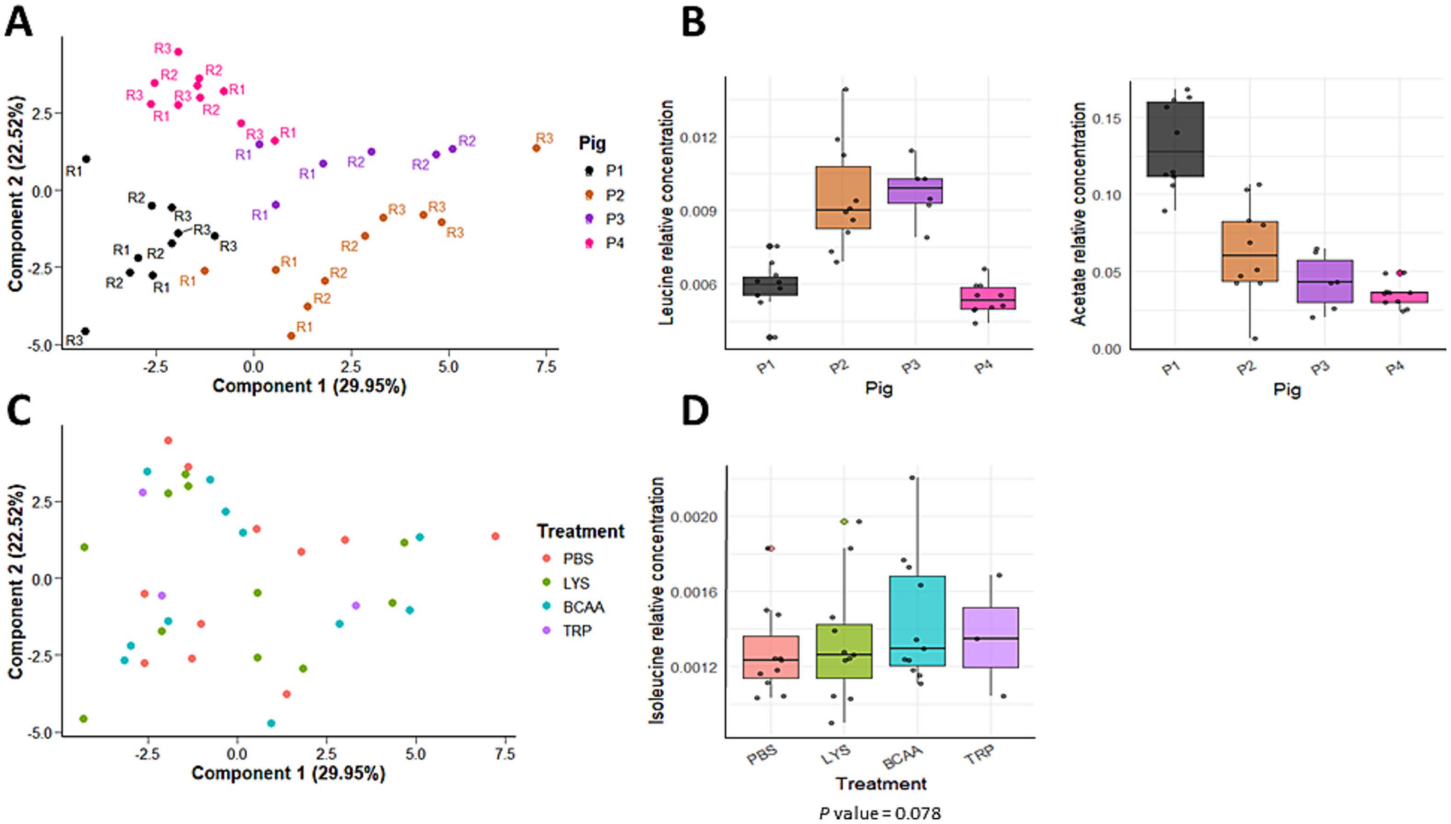
Ileal loop content metabolome. A principal component analysis (PCA) revealed that metabolome was primarily influenced by the individual pig and replicate **(A)**. Relative concentration of leucine and acetate varied among individual pigs **(B)**. AA treatments had no overall effect on the metabolome **(C)**. Isoleucine levels were influenced by treatment **(D)**. BCAA: mix of branched chain AAs including L-valine, L-leucine, and L-isoleucine; LYS: L-lysine; PBS: phosphate-buffered saline; R: replicate; TRP: L-tryptophan.

Metabolite analyses performed using the content of ileal loops are presented in Table 4 and Figures 3A–D. PCA of metabolite profiles separated individual pigs (Figure 3A). For instance, higher leucine concentration in Pig 2 and 3 and acetate concentration in Pig 1 were noted (Figure 3B), but no distinction between treatments was observed (Figures 3C,D). AA concentrations were consistent across groups (Table 4). Isoleucine levels were highest in the BCAA group, with a trend toward significance (*p* = 0.078; Table 4), likely reflecting the supplementation of branched-chain AAs in this group.

**Table 4:** Relative metabolites concentrations in porcine ileal loops contents.

Metabolites	*P-*value	Pairwise comparisons				Relative concentration (mean)				Relative concentration (SEM)			
						PBS	LYS	BCAA	TRP	PBS	LYS	BCAA	TRP
2-methylbutyrate	0.731	a	a	a	a	0.68	0.62	0.64	0.76	0.06	0.05	0.07	0.17
Acetate	0.336	a	a	a	a	65.95	76.29	72.66	68.66	14.34	13.62	15.77	23.03
Alanine	0.11	a	a	a	a	11.21	9.89	10.33	11.18	0.91	0.88	0.98	2.86
Butyrate	0.309	a	a	a	a	1.58	2.34	1.73	1.13	0.26	0.64	0.41	0.26
Choline	0.665	a	a	a	a	22.69	21.08	22.44	24.04	2.41	2.59	2.22	3.9
Creatine	0.633	a	a	a	a	46.45	49.26	47.31	33.94	7.57	7.93	8.7	6.53
Doublet@1.15 ppm	0.573	a	a	a	a	5.8	6.31	6.42	8.81	0.82	0.78	1.03	0.69
Ethanol	0.143	a	a	a	a	3.8	4.36	4.4	4.33	0.4	0.39	0.4	0.5
Fumarate	0.905	a	a	a	a	1.64	1.68	1.62	1.82	0.23	0.19	0.18	0.39
Glutamate	0.156	a	a	a	a	8.43	7.47	7.8	7.76	0.63	0.53	0.53	2.44
Glycine	0.196	a	a	a	a	33.61	27.09	30.31	30.41	2.95	2.25	2.73	5.38
Hypoxanthinne	0.611	a	a	a	a	1.31	1.25	1.16	1.27	0.1	0.12	0.07	0.11
Isobutyrate	0.634	a	a	a	a	0.86	0.98	1.07	0.97	0.13	0.13	0.15	0.14
Isoleucine	0.078	a	a	a	a	1.28	1.33	1.44	1.36	0.07	0.1	0.1	0.19
Isovalerate	0.725	a	a	a	a	0.43	0.73	0.59	0.35	0.08	0.25	0.18	0.12
Lactate	0.142	a	a	a	a	17.57	13.89	23.18	27.05	4.49	3.18	4.04	10.78
Leucine	0.434	a	a	a	a	7.52	7.59	7.41	6.52	0.78	0.78	0.72	1.22
Lysine	0.837	a	a	a	a	2.8	2.82	2.85	2.65	0.24	0.25	0.23	0.44
Methylamine	0.666	a	a	a	a	0.66	0.68	0.63	0.69	0.04	0.04	0.04	0.14
Phenylacetate	0.985	a	a	a	a	0.44	0.43	0.44	0.44	0.05	0.04	0.04	0.08
Phenylalanine	0.737	a	a	a	a	1.59	1.57	1.58	1.53	0.09	0.08	0.07	0.09
Propionate	0.857	a	a	a	a	5.11	5.39	5.4	4.69	0.44	0.57	0.44	0.6
Succinate	0.588	a	a	a	a	4.16	3.38	4.13	6.31	1.29	0.87	1.22	2.67
Trimethylamine	0.988	a	a	a	a	1.63	1.73	1.7	1.29	0.57	0.68	0.5	0.1
Tyrosine	0.938	a	a	a	a	1.27	1.25	1.29	1.32	0.07	0.07	0.06	0.08
Uracil	0.358	a	a	a	a	1.11	1.18	1.19	1.34	0.08	0.08	0.09	0.16
Valerate	0.26	a	a	a	a	0.26	0.29	0.19	0.14	0.05	0.05	0.03	0.06
Valine	0.237	a	a	a	a	3.54	3.57	3.55	2.96	0.39	0.43	0.41	0.66

**Figure 4:**
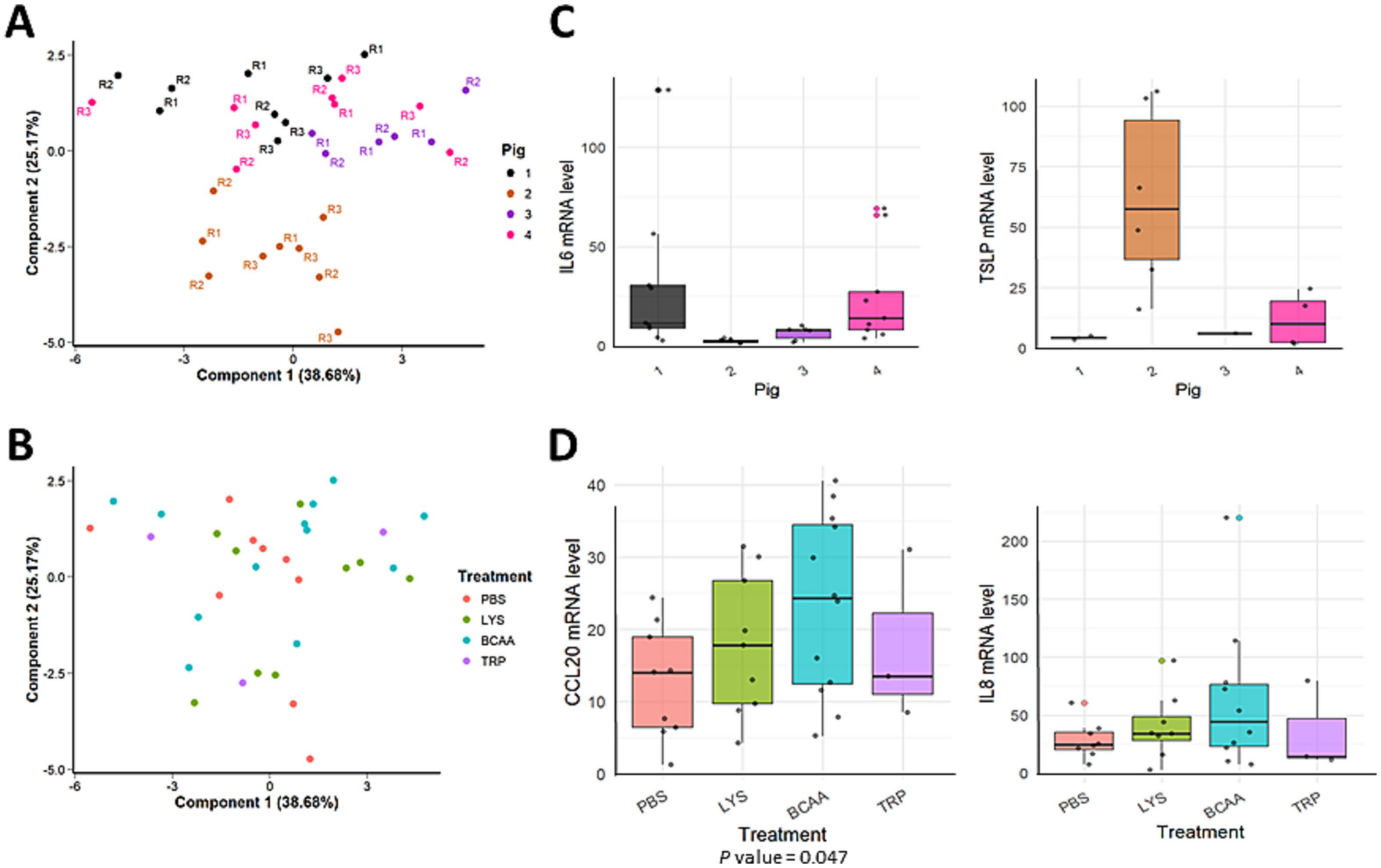
Gene expression in ileal gut wall. Principal component analysis (PCA) of transcript expression revealed that gene expression was primarily influenced by the individual pig and replicate **(A)**, while AA treatments had no overall effect **(B)**. Gene expression of IL6 and TLSP according to pigs **(C)** and CCL20 and IL8 according to treatment **(D)**. BCAA: mix of branched chain AAs including L-valine, L-leucine, and L-isoleucine; CCL20: chemokine (C-C motif) ligand 20; IL6: interleukin 6; IL8/CXCL-8: interleukin 8; LYS: L-lysine; PBS: phosphate-buffered saline; R: replicate; TSLP: thymic stromal lymphopoietin; TRP: L-tryptophan.

**Figure 5:**
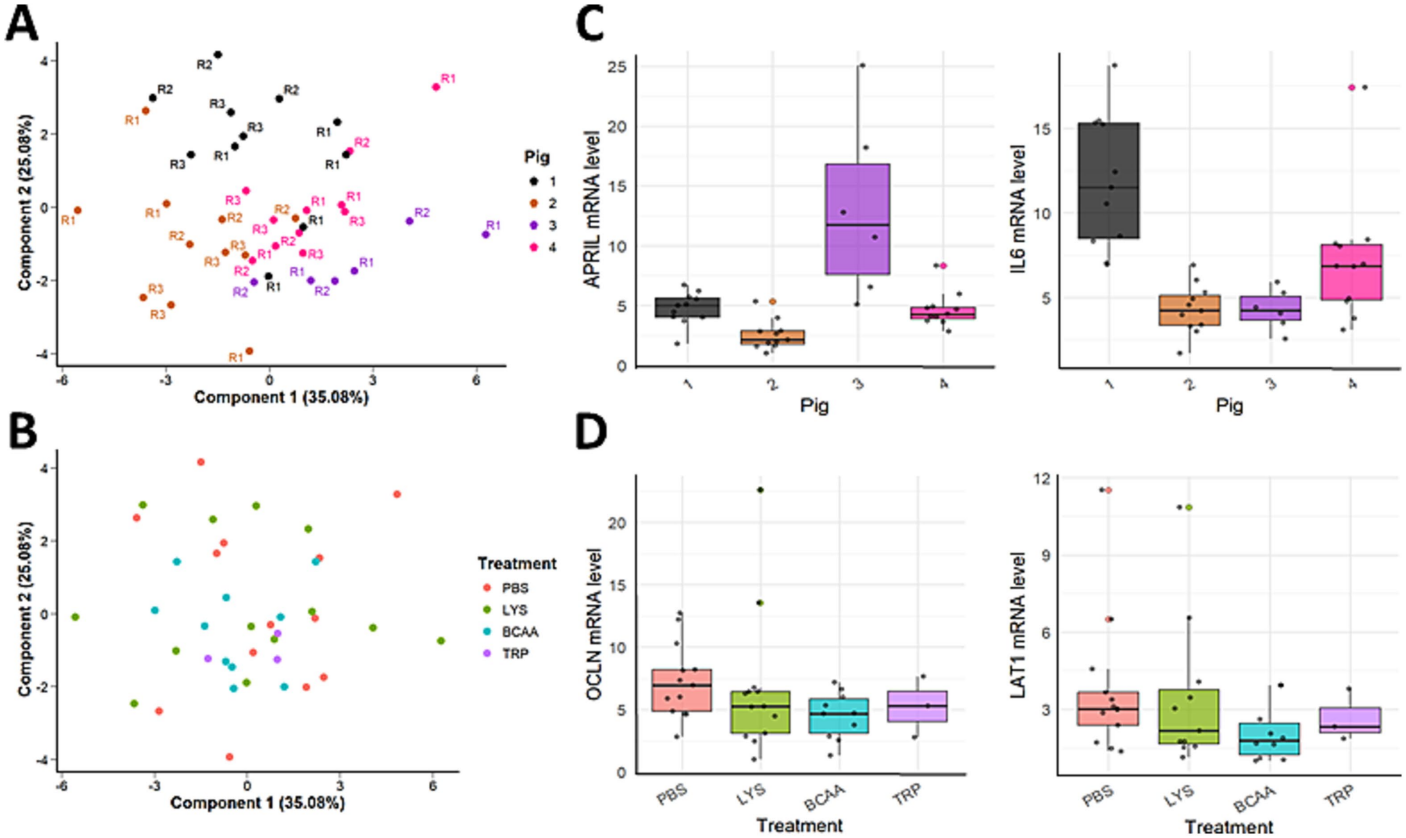
Gene expression in ileal Peyer’s patch. Principal component analysis (PCA) of transcript expression revealed that gene expression was primarily influenced by the individual pig and replicate **(A)**, whereas AA treatments had no overall effect **(B)**. Gene expression of APRIL and IL6 according to pigs **(C)** and OCLN and LAT1 according to treatment **(D)**. APRIL/TNFSF13B: A proliferation-inducing ligand; BCAA: mix of branched chain AAs including L-valine, L-leucine, and L-isoleucine; IL6: Interleukin 6; LAT1 (y^+^LAT1 or SLC7A7): large amino-acid transporter 1; LYS: L-lysine; OCLN: occludin; PBS: phosphate-buffered saline; R: replicate; TRP: L-tryptophan.

Gene expression analysis was conducted to evaluate the expression of key markers, including AA transporters (e.g., *SLC6A14* and *SLC7A9*), immune and inflammatory markers (e.g., *IL8/CXCL-8*, *CCL20*, and *CCR10*), and the tight junction protein ZO1 (for all the list see Figure 1). As expected, the two sampled tissues—the intestinal wall (Figures 4A–D and Table 5A) and Peyer’s patches (Figures 5A–D and Table 5B)—exhibited clearly distinct expression profiles for numerous transcripts, most notably for *GP2* and *OCLN* (see Supplementary Figure 2). PCA of transcript expression in the gut wall (Figures 4A,B) revealed a clear separation among the four pigs (Figure 4A) but did not distinguish between treatment groups (Figure 4B). Although no statistically significant differences were detected among the treatment groups in either the gut wall or Peyer’s patches, several trends were observed (Table 5). In the gut wall, *SLC6A14* (*ATB^0,+^*) expression tended to be higher in the branched-chain AAs (BCAA) group (*p* = 0.08; Table 5A). Similarly, *CCL20* and *IL8* showed elevated expression in the BCAA group (*p* = 0.047 and *p* = 0.061, respectively; Figure 4D and Table 5A), though these differences did not reach statistical significance when groups were compared. Examples of inter-individual variations in transcript expression were also observed, included elevated *TSLP* in the gut wall of Pig 2 (Figure 4C), increased *APRIL* in the Peyer’s patch of Pig 3 (Figure 5C), and higher *IL6* in the Peyer’s patch of Pig 1 (Figure 5C). Overall, in the Peyer’s patches, the expression profiles of the selected transcripts did not allow differentiation between the four treatments.

**Table 5:** Relative expression of genes related to inflammatory response, amino acid transport, and cellular functions in the gut wall (A) and Peyer’s patches (B).

(A) Gut wall													
Genes	*P* value	Pairwise comparisons				Relative expression (mean)				Relative expression (SEM)			
						PBS	LYS	BCAA	TRP	PBS	LYS	BCAA	TRP
*SLC7A7 (_y+_LAT1)*	0.579	a	a	a	a	5.76	7.05	6.75	8.06	1.2	1.07	0.81	2.34
*SLC6A14* *(ATB^0,+^)*	0.08	a	a	a	a	7.24	10.75	12.99	8.71	2.02	2.47	3.22	2.94
*SCL6A19 (B^0^AT1)*	0.832	a	a	a	a	73.86	101.24	66.17	82.18	20.16	42.95	25.28	73.29
*CAT-1*	0.848	a	a	a	a	18.86	22.11	26.43	19.69	5.49	5.75	12.91	13.44
*SLC7A9* *(b^0,+^AT)*	0.08	a	a	a	a	18.2	15.81	14.52	18.61	4.01	2.59	2.88	8.05
*IL6*	0.416	a	a	a	a	14.14	7.21	26.36	23.76	6.18	2.29	10.92	21.04
*IL8*	0.061	a	a	a	a	28.46	40.26	63.86	35.18	5.72	10.24	20.41	22.25
*BAFF*	0.466	a	a	a	a	4.16	4.66	6.47	4.56	0.93	0.56	1.27	1.75
*ZO1*	0.498	a	a	a	a	9074.04	8992.11	9978.2	4578.29	2527.25	809.74	3588.19	NA
*CCL20*	0.047	a	a	a	a	12.69	17.96	23.37	17.66	2.63	3.28	3.59	6.83
*OCLN*	0.117	a	a	a	a	10.13	18.56	14.7	12.76	1.94	3.62	1.96	2.58
*TSLP*	0.66	a	a	a	a	36.44	29.79	13.29	106.0	33.49	10.23	3.67	NA
*APRIL*	0.573	a	a	a	a	5.74	7.7	7.68	6.16	0.82	1.78	1.38	1.87
*NLRP6*	0.508	a	a	a	a	82.27	97.46	63.5	48.74	32.79	39.33	33.6	44.65
*CCR9*	0.539	a	a	a	a	10.84	12.55	8.33	10.22	3.42	3.13	1.79	5.96
*CCR10*	0.295	a	a	a	a	57.15	132.58	363.2	556.18	18.43	44.06	186.34	555.01
*AID*	0.885	a	a	a	a	5.58	7.7	6.04	5.59	1.32	1.44	1.33	1.98
*GP2*	0.922	a	a	a	a	3.12	3.28	4.52	3.83	0.52	0.25	1.17	1.41
*SOX8*	0.905	a	a	a	a	30.5	38.17	25.91	17.96	8.4	12.31	8.3	9.64

(B) Peyer’s patches													
Genes	*P-*value	Pairwise comparisons				Relative expression (mean)				Relative expression (SEM)			
						PBS	LYS	BCAA	TRP	PBS	LYS	BCAA	TRP
*SLC7A7 (_y+_LAT1)*	0.075	a	a	a	a	3.74	3.44	2.09	2.66	0.75	0.88	0.35	0.58
*SLC6A14* *(ATB^0,+^)*	0.778	a	a	a	a	9.86	11.21	8.5	9.12	2.01	2.4	1.9	4.19
*SCL6A19 (B^0^AT1)*	0.177	a	a	a	a	32.16	29.28	27.79	63.65	6.23	8.93	5.55	18.89
*CAT-1*	0.639	a	a	a	a	129.6	68.55	66.99	73.42	39.3	16.21	16.53	15.34
*SLC7A9* *(b^0,+^AT)*	0.132	a	a	a	a	8.1	7.03	5.42	7.59	1.76	1.42	1.03	1.23
*IL6*	0.799	a	a	a	a	7.58	7.86	6.48	5.74	1.35	1.33	1.17	1.21
*CXCL8*	0.539	a	a	a	a	24.05	27.98	16.1	11.58	5.72	10.08	4.29	2.62
*BAFF*	0.144	a	a	a	a	12.8	9.76	6.45	7.45	2.54	1.79	0.61	1.67
*ZO1*	0.379	a	a	a	a	5579.54	4626.69	7891.34	9732.59	1616.04	691.03	616.58	NA
*CCL20*	0.381	a	a	a	a	13.08	23.85	10.88	4.56	2.56	9.41	3.46	2.57
*OCLN*	0.059	a	a	a	a	7.28	6.64	4.5	5.25	0.83	1.58	0.6	1.41
*TSLP*	0.293	a	a	a	a	3.55	3.46	4.6	3.59	2.52	1.14	0.84	1.42
*APRIL*	0.202	a	a	a	a	5.45	6.46	4.03	4.93	0.89	1.96	0.56	1.15
*NLRP6*	0.182	a	a	a	a	52.56	54.12	41.48	57.05	17.14	24.2	17.13	9.54
*CCR9*	0.529	a	a	a	a	12.47	12.1	10.0	7.17	3.4	1.76	1.54	0.42
*CCR10*	0.752	a	a	a	a	769.63	728.36	418.82	188.79	490.43	414.42	144.1	73.34
*AID*	0.347	a	a	a	a	137.72	105.02	89.97	82.83	24.85	17.56	13.59	18.34
*GP2*	0.91	a	a	a	a	346.64	369.54	289.2	333.88	49.58	53.66	29.69	34.91
*SOX8*	0.444	a	a	a	a	97.97	93.21	91.89	79.26	27.61	28.3	17.31	26.24

To summarize, a few effects were observed—most notably, in the gut wall, for *CCL20* (*p* = 0.047) and to a lesser extent for *IL8* (close to significance - *p* = 0.061) (Figure 4D) where a treatment effect was detected, despite pairwise comparisons failing to reach statistical significance.

## 4 Discussion

The objective of this study was to assess the impact of individual or combined AA treatments on the porcine intestinal mucosa and microbiota using the ligated intestinal loop model (Gerdts et al., 2001). Specifically, we aimed to pioneer the use of this surgical approach to evaluate the short-term effects of AA administration within the loops, with the goal of refining and optimizing the model in future studies. This approach enables targeted evaluation of treatment effects in a confined intestinal segment while preserving blood and lymphatic connections, thereby maintaining most of the physiological integrity of the environment. The surgical procedures, which have been extensively validated (Gerdts et al., 2001; Meurens et al., 2009; Girard-Misguich et al., 2011), were successfully performed, and the pigs exhibited rapid recovery, consistent with previous observations (Bayne et al., 2025). Only one of the four pigs experienced a brief episode of diarrhea, which was quickly resolved. This brief diarrheal episode may account somewhere for the somewhat atypical behavior observed in one replicate (the third) from Pig 3. Indeed, intestinal inflammation could have influenced the microbiota and the metabolome.

The two types of collected tissues, the gut wall and the Peyer’s patch, exhibited clearly distinct expression profiles. While this observation was expected, it further validates the technical robustness of our protocol. Indeed, *GP2* is known to be highly expressed in mature M cells of Peyer’s patches (Hase et al., 2009) and the *OCLN*, the first identified tight junction protein, has major roles in the epithelial barrier and its higher expression in the gut wall segment was therefore expected (Farquhar and Palade, 1963; Furuse et al., 1993; Chelakkot et al., 2018).

Regarding the potential impact of administered AAs on the expression of selected transcripts, metabolites, and the microbiota, we failed to identify significant differences between groups, though a few near-significant trends were observed for *SLC7A7* (*^y+^LAT1*) and *OCLN* transcripts in the Peyer’s patch. The sole exception was *CCL20* transcripts in the gut wall, where an overall treatment effect was detected. CCL20 is a chemokine whose primary role is to recruit CCR6-expressing lymphocytes and dendritic cells to the mucosa (Hieshima et al., 1997). *CCL20* transcripts tended to be more highly expressed when BCAA were administered together. Similarly, *IL8* transcripts tended to more expressed in the gut wall when considering BCAA group. IL8, known alternatively as CXCL-8, is also involved in the recruitment of immune cells, and more specifically neutrophils (Snyderman et al., 1972; Matsushima et al., 2023). These observations support the idea that these BCAA may play a crucial role in strengthening the intestinal barrier, priming it to mount immune responses in which dendritic cells and lymphocytes actively participate. The absence of an effect from tryptophan treatment on the measured parameters should be interpreted cautiously, as only one tryptophan ligated loop was created per pig (limiting intra-animal replicates), while other treatments used three ligated loops.

The very limited effects of AA administration under our experimental conditions (ileal loops) could be explained by several hypotheses. First, the administered AAs were allowed to act for a relatively short period (48 h). However, we cannot rule out the possibility that the exposure time was either too long, allowing the AAs to be absorbed by the mucosa, or too short. We observed no increase in their concentration within the loop contents, nor in their metabolic derivatives. In summary, the duration of AA exposure may not have been optimal—i.e. too short or too long. Given that the transit time in the pig’s small intestine is quite short (2.3 to 4 h) (Henze et al., 2021), the exposure of the microbiota to AAs would tend to be too long. Consequently, all AAs would already be metabolized and absorbed, with little effect on metabolites. Additionally, the dose of 3 g/L may have been too low, and increasing it might facilitate the observation of significant effects. Furthermore, measuring transcript expression in whole tissue (rather than solely in the epithelium) could mask effects limited to a small number of cell types. Similarly, the tissue’s response to surgery, which was still relatively recent at the time of sample collection, may have overshadowed the cellular response to the AAs. Indeed, stimulation by AAs likely induces more subtle effects in terms of tissue response compared to infection by a pathogen such as *Salmonella Typhimurium*, where the response—even over short periods—appears quite evident (Meurens et al., 2009). Finally, inter-animal variability may have masked subtle variations in gene expression. The exposure time to AAs could be quite easily increased, as could the dose. In future experiments, more targeted approaches such as isolating epithelial cells, with or without laser microdissection, combined with single-cell analysis could be considered. The small number of animals used for a complex surgical procedure is another limitation of the study. A larger cohort with greater genetic diversity would likely increase the statistical power of our analyses (more biological replicates) while also enhancing the generalizability of our findings to the broader porcine population.

Regarding the microbiota, and as mentioned above, we did not observe any significant effects of the administered AAs. The dominant bacterial genera in the ileal loop were *Escherichia-Shigella* and *Campylobacter*, both members of the oxygen -tolerant phylum *Proteobacteria* and which is not expected at such a high abundance (more than 50%) in the healthy porcine ileum microbiota, which is normally largely dominated by *Firmicutes* (Holman et al., 2017; Ranson et al., 2025). The cessation of transit in the loops, combined with changes in intestinal lumen oxygenation, may contribute to alterations in the composition of the porcine ileal microbiota in the loop (Albenberg et al., 2014; Procházková et al., 2022). However, another study using the porcine ileal loop model did not observe such a microbiota dysbiosis after surgery (Bayne et al., 2025). In this study, and in contrast with our methods, a complex microbiota was inoculated after sterilization of the loop, and samples were collected after 5 days (*versus* 2 days in our study). These technical differences may help to further optimize the porcine gut loop model for microbiota research. Our study also lacked a microbiota analysis from the ileum outside of intestinal loops, where transit still occurs. Indeed, this less common type of control regarding the intestinal loop approach would allow for a better assessment of the effect of absent transit on the microbiota. It should be systematically included in future experiments using the gut loop approach. Another potential improvement to the procedure lies in enhancing both operative and post-operative analgesic care for the animals. Long-acting buprenorphine is now available (sustained-release buprenorphine, SRB; Zoopharm/Wedgewood, USA) (Stevey-Rindenow et al., 2025). A single subcutaneous injection (0.2 mg/kg) before the beginning of the surgery provides 72 h of analgesia, improving animal comfort while reducing the need for repeated, potentially stressful manipulations and injections. This improvement can further reduce the impact of the surgical procedure and the post-operative period on the measured biological parameters.

Overall, in the present study, using the intestinal loop surgical approach, we did not demonstrate effects of the tested AAs on the intestinal mucosa. This contrasts with previous studies showing these AAs generally have positive impacts on gut health (Wang et al., 2014; Liang et al., 2018; Zong et al., 2018; Yang and Liao, 2019; Chalvon-Demersay et al., 2021; Deng et al., 2023). However, our study also has several strengths. First, this approach offers an elegant method that more closely mimics the complexity of the whole animal compared to organoids or organ-on-a-chip models, while minimizing the number of animals used. Multiple conditions can be tested within the same animal (intra-animal replicates), in addition to inter-animal comparisons (biological replicates), in accordance with the principles of the 3Rs (Rault et al., 2022). The second strength is that our system is robust and effectively distinguishes between the intestinal wall and Peyer’s patches in terms of tissue response.

In summary, while individual pigs exhibited distinct transcript, metabolite, and microbiota profiles, no statistically significant differences were observed between AA treatment groups. However, trends for certain markers suggest potential biological effects that warrant further investigation.

## Data Availability

Sequencing reads were deposited in the National Center for Biotechnology Center for Biotechnology Information Sequence (accession number: PRJNA1308782).

## Supplementary material

SUPPLEMENTARY FIGURE 1 Interindividual variability. Non-metric dimensional analysis (NMDs) and richness of the microbiota according to pigs revealed that the microbiota is primarily influenced by the individual pig and replicate with Replicate 3 of Pig 3 being an outlier **(A)**. A principal component analysis (PCA) and relative concentration of isovalerate according to pigs revealed that the metabolome is primarily influenced by the individual pig and replicate with Replicate 3 of Pig 3 being an outlier **(B)**. Principal component analysis (PCA) of transcript expression in ileal Peyer’s patch and ileal gut wall **(C)**. P: pig; R: replicate.

SUPPLEMENTARY FIGURE 2 Gut wall *versus* Peyer’s patch. A principal component analysis (PCA) revealed that selected transcripts are differentially expressed in gut wall and Peyers’ patch samples **(A)**. Gene expression of GP2 and OCLN according to the two tissues **(B)**. GP2: glycoprotein 2; GW: gut wall; OCLN: occludin; Peyer: Peyer’s patch.

## References

[ref1] Albenberg L. Esipova T. V. Judge C. P. Bittinger K. Chen J. Laughlin A. . (2014). Correlation between intraluminal oxygen gradient and radial partitioning of intestinal microbiota. Gastroenterology 147, 1055–1063.e8. doi: 10.1053/j.gastro.2014.07.020, 25046162 PMC4252572

[ref2] Bayne J. Charavaryamath C. Hu Y. Yousefi F. Murphy M. Law A. . (2025). The swine IsoLoop model of the gut host-microbiota interface enables intra-animal treatment comparisons to advance 3R principles. Gut Microbes 17:2568706. doi: 10.1080/19490976.2025.2568706 41137517 PMC12562777

[ref3] Beaumont M. Lencina C. Painteaux L. Viémon-Desplanque J. Phornlaphat O. Lambert W. . (2022a). A mix of functional amino acids and grape polyphenols promotes the growth of piglets, modulates the gut microbiota *in vivo* and regulates epithelial homeostasis in intestinal organoids. Amino Acids 54, 1357–1369. doi: 10.1007/s00726-021-03082-9, 34642825

[ref4] Beaumont M. Roura E. Lambert W. Turni C. Michiels J. Chalvon-Demersay T. (2022b). Selective nourishing of gut microbiota with amino acids: a novel prebiotic approach? Front. Nutr. 9:66898. doi: 10.3389/fnut.2022.1066898, 36601082 PMC9806265

[ref5] Beaumont M. Vicente C. M. Plata-Calzado C. Lencina C. Jones E. Lecuelle S. . (2026). The gut microbiota metabolite isovalerate enhances the epithelial barrier function in cell monolayers derived from porcine ileum organoids. Am. J. Physiol. Gastrointest. Liver Physiol. 330, G459–G477. doi: 10.1152/ajpgi.00193.2025, 41740169

[ref6] Chalvon-Demersay T. Luise D. Le Floc’h N. Tesseraud S. Lambert W. Bosi P. . (2021). Functional amino acids in pigs and chickens: implication for gut health. Front. Vet. Sci. 8:663727. doi: 10.3389/fvets.2021.663727, 34113671 PMC8185281

[ref7] Chelakkot C. Ghim J. Ryu S. H. (2018). Mechanisms regulating intestinal barrier integrity and its pathological implications. Exp. Mol. Med. 50, 1–9. doi: 10.1038/s12276-018-0126-x, 30115904 PMC6095905

[ref8] Corl B. A. Odle J. Niu X. Moeser A. J. Gatlin L. A. Phillips O. T. . (2008). Arginine activates intestinal p70(S6k) and protein synthesis in piglet rotavirus enteritis. J. Nutr. 138, 24–29. doi: 10.1093/jn/138.1.24, 18156399

[ref9] Darmaun D. (2008). Qu’est-ce qu’un acide aminé essentiel en 2008? Nutr. Clin. Métab. 22, 142–150. doi: 10.1016/j.nupar.2008.10.007

[ref10] Deng Y. Cheng H. Li J. Han H. Qi M. Wang N. . (2023). Effects of glutamine, glutamate, and aspartate on intestinal barrier integrity and amino acid pool of the small intestine in piglets with normal or low energy diet. Front Vet Sci 10:1202369. doi: 10.3389/fvets.2023.1202369, 37576837 PMC10414990

[ref11] Escudié F. Auer L. Bernard M. Mariadassou M. Cauquil L. Vidal K. . (2018). FROGS: find, rapidly, OTUs with galaxy solution. Bioinformatics 34, 1287–1294. doi: 10.1093/bioinformatics/btx791, 29228191

[ref12] Fang X. Liu H. Liu J. Du Y. Chi Z. Bian Y. . (2025). Isobutyrate confers resistance to inflammatory bowel disease through host–microbiota interactions in pigs. Research 8:0673. doi: 10.34133/research.0673, 40342298 PMC12059313

[ref13] Farquhar M. G. Palade G. E. (1963). Junctional complexes in various epithelia. J. Cell Biol. 17, 375–412. doi: 10.1083/jcb.17.2.375, 13944428 PMC2106201

[ref14] Fayomi S. I. Erukainure O. L. Zimbili Msomi N. (2025). The essentiality of amino acids in healthiness and disease state: type II diabetes as a case study. Food Sci. Nutr. 13:e70346. doi: 10.1002/fsn3.70346, 40452790 PMC12124235

[ref15] Furuse M. Hirase T. Itoh M. Nagafuchi A. Yonemura S. Tsukita S. . (1993). Occludin: a novel integral membrane protein localizing at tight junctions. J. Cell Biol. 123, 1777–1788. doi: 10.1083/jcb.123.6.1777, 8276896 PMC2290891

[ref16] Gerdts V. Uwiera R. R. Mutwiri G. K. Wilson D. J. Bowersock T. Kidane A. . (2001). Multiple intestinal “loops” provide an *in vivo* model to analyse multiple mucosal immune responses. J. Immunol. Methods 256, 19–33. doi: 10.1016/S0022-1759(01)00429-X 11516752

[ref17] Girard-Misguich F. Cognie J. Delgado-Ortega M. Berthon P. Rossignol C. Larcher T. . (2011). Towards the establishment of a porcine model to study human amebiasis. PLoS One 6:e28795. doi: 10.1371/journal.pone.0028795, 22205970 PMC3244410

[ref18] Griebel P. J. Hein W. R. (1996). Expanding the role of Peyer’s patches in B-cell ontogeny. Immunol. Today 17, 30–39. doi: 10.1016/0167-5699(96)80566-4 8652050

[ref19] Guitton Y. Tremblay-Franco M. Le Corguillé G. Martin J.-F. Pétéra M. Roger-Mele P. . (2017). Create, run, share, publish, and reference your LC–MS, FIA–MS, GC–MS, and NMR data analysis workflows with the Workflow4Metabolomics 3.0 galaxy online infrastructure for metabolomics. Int. J. Biochem. Cell Biol. 93, 89–101. doi: 10.1016/j.biocel.2017.07.002 28710041

[ref20] Hase K. Kawano K. Nochi T. Pontes G. S. Fukuda S. Ebisawa M. . (2009). Uptake through glycoprotein 2 of FimH(+) bacteria by M cells initiates mucosal immune response. Nature 462, 226–230. doi: 10.1038/nature08529, 19907495

[ref21] He L. Yang H. Hou Y. Li T. Fang J. Zhou X. . (2013). Effects of dietary L-lysine intake on the intestinal mucosa and expression of CAT genes in weaned piglets. Amino Acids 45, 383–391. doi: 10.1007/s00726-013-1514-0, 23722415

[ref22] Henze L. J. Koehl N. J. Bennett-Lenane H. Holm R. Grimm M. Schneider F. . (2021). Characterization of gastrointestinal transit and luminal conditions in pigs using a telemetric motility capsule. Eur. J. Pharm. Sci. 156:105627. doi: 10.1016/j.ejps.2020.105627 33122007

[ref23] Hervet C. Perrin A. Renson P. Deblanc C. Muñoz M. Meurens F. . (2025). Differential impact of porcine reproductive and respiratory virus and swine influenza a virus infections on respiratory lymph nodes B cells and macrophages. Mol. Immunol. 188, 98–110. doi: 10.1016/j.molimm.2025.10.010, 41218477

[ref24] Hieshima K. Imai T. Opdenakker G. Van Damme J. Kusuda J. Tei H. . (1997). Molecular cloning of a novel human CC chemokine liver and activation-regulated chemokine (LARC) expressed in liver. Chemotactic activity for lymphocytes and gene localization on chromosome 2. J. Biol. Chem. 272, 5846–5853. doi: 10.1074/jbc.272.9.5846 9038201

[ref25] Holman D. B. Brunelle B. W. Trachsel J. Allen H. K. (2017). Meta-analysis to define a Core microbiota in the swine gut. mSystems 2, e00004–e00017. doi: 10.1128/mSystems.00004-17 28567446 PMC5443231

[ref26] Hu C. Li F. Duan Y. Yin Y. Kong X. (2019). Dietary supplementation with leucine or in combination with arginine decreases body fat weight and alters gut microbiota composition in finishing pigs. Front. Microbiol. 10:1767. doi: 10.3389/fmicb.2019.01767, 31456756 PMC6700229

[ref27] Kong S. Zhang Y. H. Zhang W. (2018). Regulation of intestinal epithelial cells properties and functions by amino acids. Biomed. Res. Int. 2018, 1–10. doi: 10.1155/2018/2819154, 29854738 PMC5966675

[ref28] Koo B. Yang C. Nyachoti C. M. (2025). Effects of functional amino acid blend dietary supplementation with different feeding regimens on growth performance and protein utilization in weaned pigs. Can. J. Anim. Sci. 105, 1–11. doi: 10.1139/cjas-2023-0128

[ref29] Larcher T. Fablet C. Renson P. Ménard D. Hervet C. Saade G. . (2019). Assessment of pulmonary tissue responses in pigs challenged with PRRSV Lena strain shows better protection after immunization with field than vaccine strains. Vet. Microbiol. 230, 249–259. doi: 10.1016/j.vetmic.2019.01.022, 30827397

[ref30] Liang H. Dai Z. Liu N. Ji Y. Chen J. Zhang Y. . (2018). Dietary L-tryptophan modulates the structural and functional composition of the intestinal microbiome in weaned piglets. Front. Microbiol. 9:1736. doi: 10.3389/fmicb.2018.01736, 30131777 PMC6090026

[ref31] Ling Z.-N. Jiang Y.-F. Ru J.-N. Lu J.-H. Ding B. Wu J. (2023). Amino acid metabolism in health and disease. Sig Transduct Target Ther 8:345. doi: 10.1038/s41392-023-01569-3, 37699892 PMC10497558

[ref32] Mao X. Gu C. Ren M. Chen D. Yu B. He J. . (2018). L-isoleucine administration alleviates rotavirus infection and immune response in the weaned piglet model. Front. Immunol. 9:1654. doi: 10.3389/fimmu.2018.01654, 30061901 PMC6054962

[ref33] Mao X. Liu M. Tang J. Chen H. Chen D. Yu B. . (2015). Dietary leucine supplementation improves the mucin production in the Jejunal mucosa of the weaned pigs challenged by porcine rotavirus. PLoS One 10:e0137380. doi: 10.1371/journal.pone.0137380, 26336074 PMC4559446

[ref34] Matsushima K. Shichino S. Ueha S. (2023). Thirty-five years since the discovery of chemotactic cytokines, interleukin-8 and MCAF: a historical overview. Proc. Jpn. Acad. Ser. B Phys. Biol. Sci. 99, 213–226. doi: 10.2183/pjab.99.014, 37518010 PMC10700015

[ref35] Metzler-Zebeli B. U. Lawlor P. G. Magowan E. Zebeli Q. (2018). Interactions between metabolically active bacteria and host gene expression at the cecal mucosa in pigs of diverging feed efficiency. J. Anim. Sci. 96, 2249–2264. doi: 10.1093/jas/sky118, 29746643 PMC6095344

[ref36] Meurens F. Berri M. Auray G. Melo S. Levast B. Virlogeux-Payant I. . (2009). Early immune response following *Salmonella enterica* subspecies *enterica* serovar typhimurium infection in porcine jejunal gut loops. Vet. Res. 40:05. doi: 10.1051/vetres:2008043, 18922229 PMC2695014

[ref37] Miner-Williams W. Moughan P. J. Fuller M. F. (2009). Endogenous components of digesta protein from the terminal ileum of pigs fed a casein-based diet. J. Agric. Food Chem. 57, 2072–2078. doi: 10.1021/jf8023886, 19203191

[ref38] Mou Q. Yang H.-S. Yin Y.-L. Huang P.-F. (2019). Amino acids influencing intestinal development and health of the piglets. Animals 9:302. doi: 10.3390/ani9060302, 31159180 PMC6617173

[ref39] Mutwiri G. Watts T. Lew L. Beskorwayne T. Papp Z. Baca-Estrada M. E. . (1999). Ileal and jejunal Peyer’s patches play distinct roles in mucosal immunity of sheep. Immunology 97, 455–461. doi: 10.1046/j.1365-2567.1999.00791.x, 10447767 PMC2326853

[ref40] Nygard A. B. Jorgensen C. B. Cirera S. Fredholm M. (2007). Selection of reference genes for gene expression studies in pig tissues using SYBR green qPCR. BMC Mol. Biol. 8:67. doi: 10.1186/1471-2199-8-67, 17697375 PMC2000887

[ref41] Oliphant K. Allen-Vercoe E. (2019). Macronutrient metabolism by the human gut microbiome: major fermentation by-products and their impact on host health. Microbiome 7:91. doi: 10.1186/s40168-019-0704-8 31196177 PMC6567490

[ref42] Paës C. Gidenne T. Bébin K. Duperray J. Gohier C. Guené-Grand E. . (2020). Early introduction of solid feeds: ingestion level matters more than prebiotic supplementation for shaping gut microbiota. Front. Vet. Sci. 7:261. doi: 10.3389/fvets.2020.00261 32478111 PMC7242618

[ref43] Pearce S. C. Nisley M. J. Kerr B. J. Sparks C. Gabler N. K. (2024). Effects of dietary protein level on intestinal function and inflammation in nursery pigs. J. Anim. Sci. 102:skae077. doi: 10.1093/jas/skae077, 38504643 PMC11015048

[ref44] Procházková N. Falony G. Dragsted L. O. Licht T. R. Raes J. Roager H. M. (2022). Advancing human gut microbiota research by considering gut transit time. Gut 72, 180–191. doi: 10.1136/gutjnl-2022-328166, 36171079 PMC9763197

[ref45] Ranson A. Vazquez Gomez M. Alili R. Durrafourd J. Vitalis O. Taillandier P. . (2025). Moderate increase in dietary fat induces alterations of microbiota and metabolome along the digestive tract prior to systemic metabolic changes: insights from a pig model. Gut Microbes 17:2587964. doi: 10.1080/19490976.2025.2587964, 41327907 PMC12674348

[ref46] Rault J.-L. Binder R. Grimm H. (2022). Rethink farm animal production: the 3Rs. Science 378, 842–842. doi: 10.1126/science.adf3351, 36423289

[ref47] Read T. Fortun-Lamothe L. Pascal G. Le Boulch M. Cauquil L. Gabinaud B. . (2019). Diversity and co-occurrence pattern analysis of Cecal microbiota establishment at the onset of solid feeding in young rabbits. Front. Microbiol. 10:973. doi: 10.3389/fmicb.2019.00973, 31134019 PMC6524096

[ref48] Roager H. M. Licht T. R. (2018). Microbial tryptophan catabolites in health and disease. Nat. Commun. 9:3294. doi: 10.1038/s41467-018-05470-4, 30120222 PMC6098093

[ref49] Saade G. Ménard D. Hervet C. Renson P. Hue E. Zhu J. . (2020). Porcine reproductive and respiratory syndrome virus interferes with swine influenza a virus infection of epithelial cells. Vaccines (Basel) 8:1–22. doi: 10.3390/vaccines8030508, 32899579 PMC7565700

[ref50] Snyderman R. Altman L. C. Hausman M. S. Mergenhagen S. E. (1972). Human mononuclear leukocyte chemotaxis: a quantitative assay for humoral and cellular chemotactic factors. J. Immunol. 108, 857–860. doi: 10.4049/jimmunol.108.3.857, 5011759

[ref51] Stevey-Rindenow L. M. Saenz M. La V. Franklin C. Aycock-Williams A. Fueger P. T. (2025). Pharmacokinetics of extended-release buprenorphine in female Yorkshire swine (*Sus scrofa* domestica). Am. J. Vet. Res. 86:ajvr.24.10.0313. doi: 10.2460/ajvr.24.10.0313, 39983311

[ref52] Sun Y. Wu Z. Li W. Zhang C. Sun K. Ji Y. . (2015). Dietary L-leucine supplementation enhances intestinal development in suckling piglets. Amino Acids 47, 1517–1525. doi: 10.1007/s00726-015-1985-2, 25940921

[ref53] Tang Y. Tan B. Li G. Li J. Ji P. Yin Y. (2018). The regulatory role of MeAIB in protein metabolism and the mTOR signaling pathway in porcine enterocytes. Int. J. Mol. Sci. 19:714. doi: 10.3390/ijms19030714, 29498661 PMC5877575

[ref54] Van den Abbeele P. Ghyselinck J. Marzorati M. Koch A.-M. Lambert W. Michiels J. . (2022). The effect of amino acids on production of SCFA and bCFA by members of the porcine colonic microbiota. Microorganisms 10:762. doi: 10.3390/microorganisms10040762, 35456812 PMC9025589

[ref55] Vandesompele J. De Preter K. Pattyn F. Poppe B. Van Roy N. De Paepe A. . (2002). Accurate normalization of real-time quantitative RT-PCR data by geometric averaging of multiple internal control genes. Genome Biol. 3:RESEARCH0034. doi: 10.1186/gb-2002-3-7-research0034, 12184808 PMC126239

[ref56] Wang W. W. Qiao S. Y. Li D. F. (2009). Amino acids and gut function. Amino Acids 37, 105–110. doi: 10.1007/s00726-008-0152-4 18670730

[ref57] Wang W. Wu Z. Lin G. Hu S. Wang B. Dai Z. . (2014). Glycine stimulates protein synthesis and inhibits oxidative stress in pig small intestinal epithelial cells. J. Nutr. 144, 1540–1548. doi: 10.3945/jn.114.194001, 25122646

[ref58] Xiong X. Tan B. Song M. Ji P. Kim K. Yin Y. . (2019). Nutritional intervention for the intestinal development and health of weaned pigs. Front. Vet. Sci. 6:46. doi: 10.3389/fvets.2019.00046, 30847348 PMC6393345

[ref59] Yang Z. Liao S. F. (2019). Physiological effects of dietary amino acids on gut health and functions of swine. Front. Vet. Sci. 6:169. doi: 10.3389/fvets.2019.00169, 31245390 PMC6579841

[ref60] Zong E. Huang P. Zhang W. Li J. Li Y. Ding X. . (2018). The effects of dietary sulfur amino acids on growth performance, intestinal morphology, enzyme activity, and nutrient transporters in weaning piglets1. J. Anim. Sci. 96, 1130–1139. doi: 10.1093/jas/skx003, 29373684 PMC6093572

