# Supplementary material for: Porcine gut loops to explore the impact of amino acids on the host–microbiota crosstalk in the ileum
Source: Front Microbiol. 2026 Jun 5;17:1854850. doi: 10.3389/fmicb.2026.1854850 (PMC13278901; doi:10.3389/fmicb.2026.1854850)

Supplementary Figure 1

A

Ileal microbiota

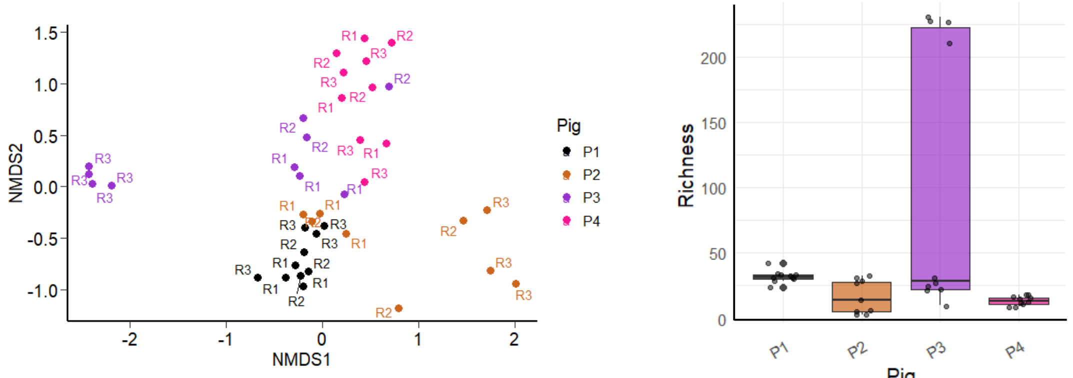

Pig P-value < 0.001, $R^2 = 0.51$

Replicate P-value = 0.306, $R^2 = 0.06$

B

Ileal metabolome

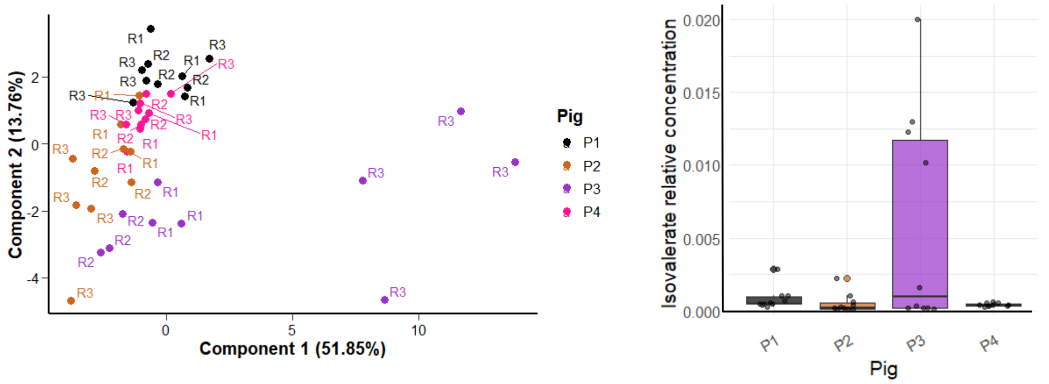

C

Gene expression in ileal Peyer's patch

Gene expression in ileal gut wall

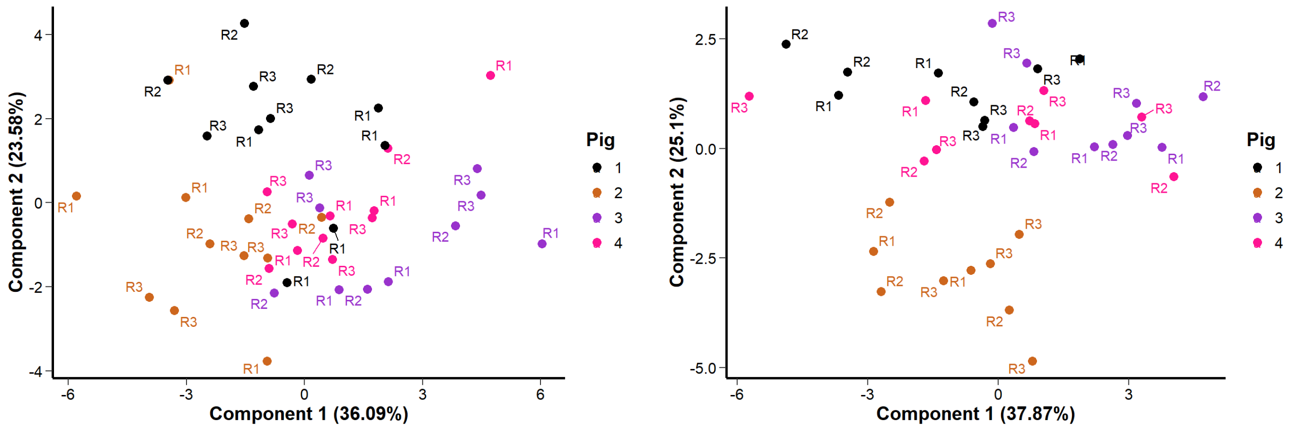

Supplementary Figure 2

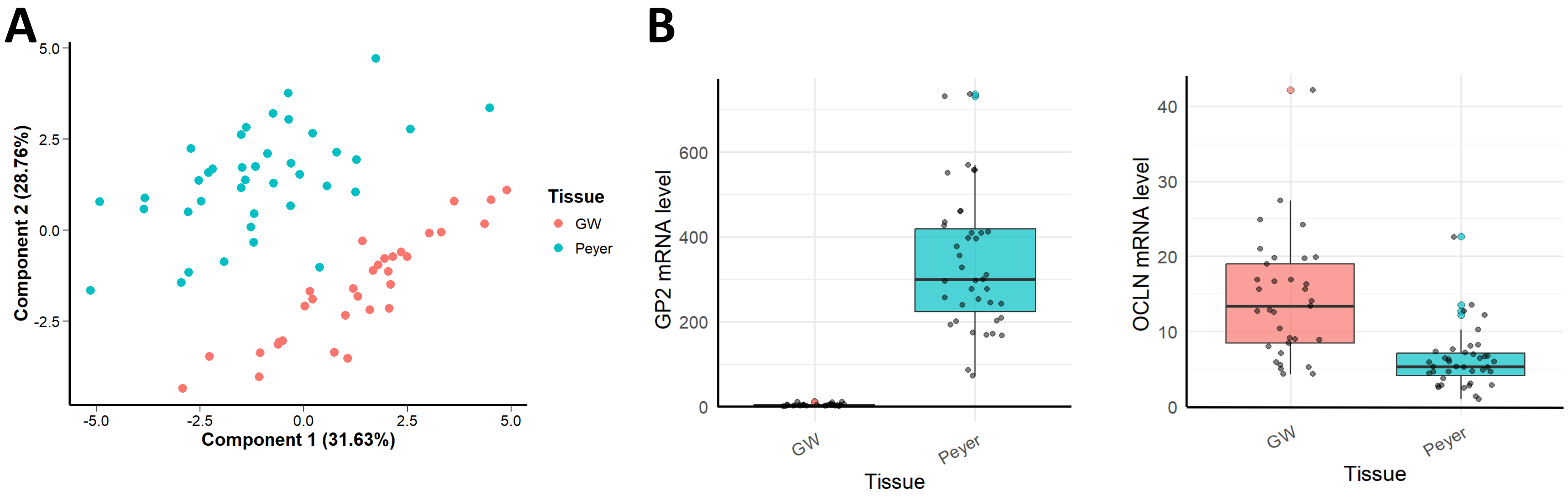

Supplement: SUPPLEMENTARY FIGURE 1 — Interindividual variability. Non-metric dimensional analysis (NMDs) and richness of the microbiota according to pigs revealed that the microbiota is primarily influenced by the individual pig and replicate with Replicate 3 of Pig 3 being an outlier (A). A principal component analysis (PCA) and relative concentration of isovalerate according to pigs revealed that the metabolome is primarily influenced by the individual pig and replicate with Replicate 3 of Pig 3 being an outlier (B). Principal component analysis (PCA) of transcript expression in ileal Peyer’s patch and ileal gut wall (C). P: pig; R: replicate. [file Data_Sheet_1.PDF]
